# Can drug-induced sleep endoscopy improve the success rates of tongue base surgery?

**DOI:** 10.1186/s40463-020-00405-w

**Published:** 2020-02-24

**Authors:** Jong-Gyun Ha, Youngwoo Lee, Jae Sung Nam, Jeong Jin Park, Joo-Heon Yoon, Chang-Hoon Kim, Hyung-Ju Cho

**Affiliations:** 1 Department of Otorhinolaryngology, Yonsei University College of Medicine, 50-1 Yonsei-ro, Seodaemun-gu, Seoul, 03722 South Korea; 2 The Airway Mucus Institute, Yonsei University College of Medicine, Seoul, South Korea; 3 Korea Mouse Sensory Phenotyping Center, Yonsei University College of Medicine, Seoul, South Korea; 4 Medical Research Center, Yonsei University College of Medicine, Seoul, South Korea

**Keywords:** Obstructive sleep apnea, Drug-induced sleep endoscopy, Muller maneuver, Tongue base surgery

## Abstract

**Background:**

The purpose of this study was to determine the therapeutic value of drug-induced sleep endoscopy (DISE) by comparing the outcomes of tongue base surgery based on Muller’s maneuver (MM) and those based on DISE in obstructive sleep apnea (OSA) patients.

**Methods:**

Ninety-five patients who underwent the tongue base surgery in combination with palatal surgery for OSA at a tertiary referral hospital between March 2012 and March 2019 were enrolled in this retrospective comparative study. Forty-seven patients underwent MM for surgical decision and 48 patients underwent DISE in addition to MM for surgical decision. Surgical success was defined according to the Sher criteria (postoperative apnea-hypopnea index [AHI] < 20/h and ≥ 50% reduction in preoperative AHI), and AHI improvement (%) was defined as (preoperative AHI-postoperative AHI) × 100/preoperative AHI. For comparison between the MM and DISE groups, *p*-values were calculated using independent or paired t-tests for continuous variables and using chi-square test for categorical variables.

**Results:**

By comparing the results of MM and DISE, consensus on the tongue base level showed insignificant concordance (kappa = 0.017, *p* = 0.865), whereas that on the oropharynx level showed fair agreement (kappa =0.241, *p* = 0.005). AHI, supine AHI, rapid eyeball movement (REM) AHI, non-REM AHI, and nadir oxygen saturation were all significantly improved after the tongue base surgery in both groups. The MM group showed a significant improvement in the Epworth sleepiness scale after the tongue base surgery (*p* = 0.014), whereas the DISE group did not (*p* = 0.165). However, there was no significant difference in the AHI improvement (MM group = 47.0 ± 32.0, DISE group = 48.3 ± 35.4, *p* = 0.852) and surgical success (MM group = 42.6%, DISE group = 45.8%, *p* = 0.748) between the groups. Tonsil grade (*p* < 0.05) and occlusion at the oropharynx lateral wall (*p* = 0.031) were significantly related to surgical success in the MM group.

**Conclusions:**

In the judgment of the tongue base surgery, MM and DISE findings showed poor agreement. DISE might affect the surgical decision on the tongue base surgery in OSA patients; however, there was a lack of evidence regarding the superiority of DISE over MM with respect to the surgical outcomes.

## Background

Obstructive sleep apnea (OSA) is a syndrome characterized by repetitive episodes of complete or partial collapse of the upper airway during sleep resulting in cessation or reduction of airflow and significant oxygen desaturation [1]. In general, the optimal treatment method for OSA is determined according to the patient’s anatomical structures (tongue base, tonsil, soft palate, adenoid, nasal septum, and inferior turbinate), polysomnographic results, and personal preferences [2]. Continuous positive airway pressure (CPAP) therapy can reduce the risk of cardiovascular comorbidity in OSA patients [3] and is considered the first therapeutic option for OSA patients. However, other therapeutic options, including surgery, are considered for CPAP-intolerant OSA patients [4].

Pre-operative physical evaluation with nasopharyngoscopy is useful to determine the level or pattern of airway collapse. Traditional nasopharyngoscopic examination of the upper airway is generally performed during the awake state. Muller’s maneuver (MM) is an established and non-invasive flexible endoscopic technique that can be performed easily at an outpatient clinic while the patient is awake [5]. However, the technique does not reflect the actual status of the upper airway during the sleep state of a patient. Since Croft and Pringle introduced drug-induced sleep endoscopy (DISE) in 1991, it has been used widely to evaluate the upper airway under similar conditions to sleeping status [6]. Several studies have attempted to compare the results of DISE with those of awake examination performed via MM. According to recent studies, the relation of obstruction of the lateral wall and that at the retropalatal level showed relatively high conformity between MM and DISE; however, obstruction at the tongue base level showed a lower consensus [7, 8]. In some recent studies, about 40–50% of the surgical plan based on awake examination changed after performing DISE, especially at the tongue base level [9, 10]. Therefore, DISE may be more effective and efficient for evaluating obstruction at the tongue base level in OSA patients than conventional MM. However, it remains unclear whether surgical treatment based on DISE yields better results than that based on MM.

Therefore, the purpose of this study was to determine the therapeutic value of DISE by comparing the outcomes of tongue base surgery in OSA patients based on the findings of MM and DISE.

## Methods

### Patient profiles

From January 2013 to March 2019, we retrospectively reviewed the medical records of patients who underwent sleep surgery for OSA at Severance Hospital, a tertiary referral hospital in South Korea. Inclusion criteria for patients were as follows: (1) age ≥ 19 years (2); diagnosed with OSA (apnea-hypopnea index [AHI] ≥ 5/h) using overnight polysomnography (PSG) (3); non-compliant or refused CPAP therapy, as a nonsurgical treatment (4); underwent tongue base surgery (e.g., tongue base resection using a coblator or the da Vinci robot) with or without nasal surgery (e.g., septoplasty, turbinoplasty) for OSA improvement (5); underwent preoperative nasopharyngoscopic examinations, such as MM and/or DISE; and (6) underwent postoperative PSG at 3 months or later. Patients with a history of previous airway surgery such as uvulopalatopharyngoplasty (UPPP), lateral pharyngoplasty (LP), or tonsillectomy prior to standard PSG were excluded.

Of the 137 patients who underwent sleep surgery during the period, 36 were excluded because they underwent palatal surgery (such as UPPP and LP) without the tongue base resection. Six patients were additionally excluded because of a history of previous airway surgery. Consequently, 95 OSA patients who underwent multilevel palate and tongue base surgery were included in this study.

All patients were divided into two groups according to their airway evaluation methods. Forty-seven patients comprised the MM group who underwent only MM for surgical indication, whereas 48 patients comprised the DISE group who underwent both MM and DISE.

### Airway evaluation

All patients underwent upper airway evaluation using the Friedman staging system [11] and modified Mallampati grading [12]. Preoperative nasopharyngoscopic examination with MM and/or DISE in the supine position was performed for surgical decision [13, 14]. MM was performed basically for all patients prepared for sleep apnea surgery. However, if the results of the MM test were ambiguous for making surgical decisions or there was a mismatch between MM and PSG (e.g., in severe obstructive apnea without tongue base occlusion on MM), the DISE test was further recommended and performed by a single surgeon (Dr. H.-J Cho). Patients aged > 60 years or those who did not wish to undergo additional sedation testing did not undergo DISE, whereas some patients declined the test due to financial reasons.

DISE procedures were performed in the operating theater, whereas MM was performed at the outpatient clinic. Propofol alone, propofol-remifentanil combination, or dexmedetomidine-remifentanil combination was infused for inducing sleep apnea with proper monitoring of the participant according to the protocol, as reflected in our previous report [15].

The findings of MM and DISE were assessed using the modified VOTE classification system as suggested by Kezirian et al. [16]. The site and character of obstruction of the anatomical structure were assessed as follows: the velum, oropharyngeal lateral wall, tongue base, and epiglottis. The degree of occlusion, as revealed using nasopharyngoscopy, was categorized as 0 point = no obstruction (0–25%), 1 point = partial occlusion (25–75%), and 2 points = complete occlusion (≥76%).

### Surgical decision protocol and surgical techniques

Surgical decision for the tongue base surgery in the MM group was followed to the results of MM and that in the DISE group was followed to the results of DISE. Specific surgical decision protocol followed is as follows: nasal surgery, including septoplasty and turbinoplasty, was performed for patients with a deviated nasal septum and/or hypertrophied inferior turbinate found on nasal endoscopy and/or CT scan. Overlapping LP was performed for patients when the score of velum was > 1 with a pattern of concentric or A-P/lateral wall obstruction on DISE and/or MM. Endoscopic coblator-assisted tongue base resection or transoral robotic tongue base resection was performed for patients who showed partial or complete obstruction at the tongue base level (MM or DISE score ≥ 1). Surgical techniques of overlapping LP, endoscopic coblator-assisted tongue base resection, and transoral robotic tongue base resection were well described in our previous study [17]. Consequently, surgical procedure for tongue base resection was electively performed using a coblator or the da Vinci robot, with other palatal surgery (e.g., LP) and/or nasal surgery (e.g., septoplasty, turbinoplasty).

### Outcome measurement

All patients underwent pre- and postoperative (at least 3 months later) standard PSG (Comet-PLUS® XL, Grass Technologies, Warwick, RI, USA). Response rate was assessed based on three definitions: [1] AHI < 5/h (complete resolution) [2]; AHI < 20/h with ≥50% AHI improvement (Sher criteria) [18]; and [3] ≥ 50% AHI improvement. The surgical success was defined according to the Sher criteria (definition 2). Additionally, AHI improvement (%) was defined as (preoperative AHI − postoperative AHI) × 100/preoperative AHI.

### Statistical analyses

All continuous data are reported as mean ± standard deviation. For comparison between the MM and DISE groups, *p*-values were calculated using independent or paired t-tests for continuous variables and using chi-square test for categorical variables. IBM SPSS version 23.0 statistical software (IBM Corp., Armonk, N.Y., USA) was used for statistical analysis of data. *p* < 0.05 was considered statistically significant.

## Results

### Patient profiles

The demographic characteristics of participants are shown in Table 1. The mean age of patients in the MM group was 44.3 ± 12.1 years and that of patients in the DISE group was 41.8 ± 12.2 years (*p* = 0.308). Both groups were male-predominant (MM group, 85.1%; DISE group, 87.5%, *p* = 0.734). The mean body mass index (BMI) of patients in the MM group (25.3 ± 3.2 kg/m^2^) was lower than that of patients in the DISE group (26.3 ± 3.2 kg/m^2^); however, the difference was not statistically significant (*p* = 0.130). There was also no statistically significant difference with respect to the tonsil grade (*p* = 0.076), Friedman staging (*p* = 0.056), and modified Mallampati grading (*p* = 0.570) between the two groups.

**Table 1:** Demographic characteristics of participants (*n* = 95)

	MM group (*n* = 47)	DISE group (*n* = 48)	*P-*value
Age, years	44.3 ± 12.1	41.8 ± 12.2	0.308
Sex, n (%)			0.734
Male	40 (85.1%)	42 (87.5%)	
Female	7 (14.9%)	6 (12.5%)	
BMI, kg/m^2^	25.3 ± 3.2	26.3 ± 3.2	0.130
NC	38.7 ± 8.6	38.8 ± 2.9	0.957
W/H ratio	0.9 ± 0.1	0.9 ± 0.1	0.375
Tonsil	1.5 ± 0.8	1.8 ± 0.9	0.076
MMP	2.9 ± 0.7	3.0 ± 0.8	0.570
Freidman stage	2.6 ± 0.6	2.3 ± 0.6	0.056

All data, except sex, are represented as mean ± standard deviation

Independent t-test was performed

*Abbreviations*: *MM* Muller’s maneuver, *DISE* Drug-induced sleep endoscopy, *BMI* Body mass index, *NC* Neck circumference, *W/H ratio* Waist/hip ratio, *MMP* Modified Mallampati grade

All participants in both groups underwent multilevel surgery of palatal surgery and tongue base surgery. For palatal surgery, LP was mainly performed in both groups (MM group 85.1%, DISE group 100%, *p* = 0.005). Endoscopic coblator-assisted tongue base surgery was performed more than transoral robotic tongue base surgery in both groups (MM group 70.2%, DISE group 81.3%, *p* = 0.209). About 60% of the participants in the MM group underwent nasal surgery (septoplasty 51.1%, turbinoplasty 63.8%), whereas less than half of those in the DISE group underwent nasal surgery (septoplasty 37.5%, turbinoplasty 45.8%). However, there was no significant difference in nasal surgery performed between both groups (*p* = 0.078, data not shown).

### Comparison of MM and DISE findings

Results of MM and DISE of each patient in the DISE group were compared to identify their agreement for evaluation of the degree and pattern of obstruction at each anatomical level of the upper airway (Table 2). This comparison revealed fair agreement for the oropharyngeal lateral wall (kappa = 0.241, *p* = 0.005), but did not show significant concordance at the velum, tongue base, and epiglottis. In 27 patients (56.3%), the degree of obstruction at the tongue base level assessed by DISE was greater than that assessed by MM, and in 19 patients (39.6%), the results were the same. In only two patients (4.2%), the degree of obstruction assessed by MM was higher than that assessed by DISE.

**Table 2:** Concordance between MM and DISE at the anatomic levels (DISE group, *n* = 48)

Obstruction level	MM 0	MM 1	MM 2	kappa	*P-*value
	DISE 0	DISE 1	DISE 2		
V	0 (0%)	20 (41.7%)	28 (58.3%)	0.115	0.087
	0 (0%)	2 (4.2%)	46 (95.8%)		
O	3 (62.5%)	32 (66.7%)	12 (25.0%)	0.241	0.005**
	7 (14.6%)	13 (27.1%)	28 (58.3%)		
T	11 (22.9%)	32 (66.7%)	5 (10.4%)	0.017	0.865
	0 (0%)	24 (50.0%)	24 (50.0%)		
E	44 (91.7%)	2 (4.2%)	1 (2.1%)	0.122	0.202
	39 (81.3%)	3 (6.3%)	5 (10.4%)		

The degree of occlusion as per the nasopharyngoscopic study: 0 = no obstruction (0–25%), 1 = partial occlusion (25–75%), and 2 = complete occlusion (≥76%)

Interpretation of kappa index: < 0, no agreement; 0–0.19, poor agreement; 0.20–0.39, fair agreement; 0.40–0.59, moderate agreement; 0.60–0.79, substantial agreement; 0.80–1.00, almost perfect agreement

*Abbreviations*: *MM* Muller’s maneuver, *DISE* Drug-induced sleep endoscopy, *V* Velum, *O* Oropharynx lateral wall, *T* Tongue base, *E* Epiglottis

* *p* < 0.05, **p < 0.01, ***p < 0.001; statistical analyses were performed using Cohen’s Kappa test

To compare the degree of occlusion between MM and DISE, we also compared the mean difference between the two methods in the DISE group using paired t-test (Table 3). All anatomical levels, except the epiglottis, showed significant mean differences between MM and DISE. The mean difference between MM and DISE was the greatest at the tongue base level (mean difference − 0.63, 95% CI − 0.83 to − 0.42, *p* < 0.001).

**Table 3:** Comparison of occlusion severity between MM and DISE according to the anatomic levels (DISE group, *n* = 48)

Obstruction level	Nasopharyngoscopic study		Mean difference (95% CI)	*P-*value
	MM	DISE		
V	1.58 ± 0.50	1.96 ± 0.20	−0.38 (− 0.52, − 0.23)	< 0.001***
O	1.19 ± 0.53	1.44 ± 0.74	− 0.25 (− 0.44, − 0.06)	0.013*
T	0.92 ± 0.54	1.54 ± 0.50	− 0.63 (− 0.83, − 0.42)	< 0.001***
E	0.09 ± 0.35	0.28 ± 0.65	− 0.19 (− 0.41, − 0.03)	0.083

Data are represented as mean ± standard deviation

*Abbreviations*: *MM* Muller’s maneuver, *DISE* Drug-induced sleep endoscopy,*CI* Confidence interval), *V* Velum, *O* Oropharynx lateral wall, *T* Tongue base, *E* Epiglottis

* *p* < 0.05, **p < 0.01, ***p < 0.001; statistical analyses were performed using paired t-test

### Comparison of surgical outcomes between the MM and DISE groups

Table 4 shows the results of pre- and postoperative PSG and Epworth sleepiness scale (ESS) scores in both groups. AHI, supine AHI, non-rapid eyeball movement AHI (AHI_NREM_), rapid eyeball movement AHI (AHI_REM_), and nadir oxygen saturation (nadir O_2_ sat) were all significantly improved after the multilevel surgery in both groups. However, the ESS score was not significantly improved in the DISE group (preoperative vs. postoperative = 8.1 ± 5.2 vs. 7.4 ± 5.0, *p* = 0.165), whereas it significantly improved in the MM group (9.4 ± 4.9 vs. 7.7 ± 4.1, *p* = 0.014).

**Table 4:** Evaluation of the pre- and postoperative polysomnographic findings and ESS scores in both groups

	MM group (*n* = 47)			DISE group (*n* = 48)		
	Pre Op	Post Op	*P-*value	Pre Op	Post Op	*P-*value
AHI	39.7 ± 18.8	20.0 ± 13.9	< 0.001***	48.1 ± 20.7	24.3 ± 20.1	< 0.001***
Supine AHI	53.5 ± 22.5	32.1 ± 22.9	0.002**	62.0 ± 21.5	32.3 ± 25.3	< 0.001***
AHI_NREM_	39.3 ± 19.2	19.2 ± 14.0	< 0.001***	49.8 ± 22.2	24.0 ± 21.2	< 0.001***
AHI_REM_	36.2 ± 22.4	18.7 ± 13.3	< 0.001***	50.4 ± 25.5	24.5 ± 20.2	< 0.001***
Nadir O_2_ saturation (%)	80.3 ± 7.0	83.3 ± 8.3	0.018*	77.7 ± 9.1	84.3 ± 7.0	< 0.001***
ESS	9.4 ± 4.9	7.7 ± 4.1	< 0.014*	8.1 ± 5.2	7.4 ± 5.0	0.165

Data are represented as mean ± standard deviation

*Abbreviations*: *MM* Muller’s maneuver, *DISE* Drug-induced sleep endoscopy, *AHI* Apnea-hypopnea index, *NREM* Non-rapid eye movement sleep, *REM* Rapid eye movement sleep, *ESS* Epworth sleepiness scale

* *p* < 0.05, **p < 0.01, ***p < 0.001; statistical analyses were performed using paired t-test

**Fig. 1:**
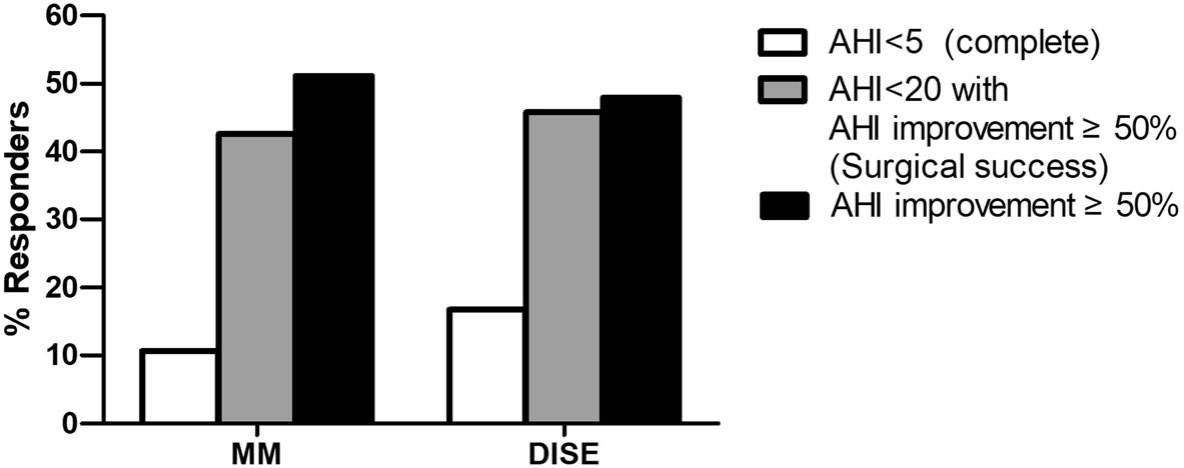
Comparison of response rates after tongue base surgery according to MM and DISE. Response rate was assessed based on three definitions: [1] AHI < 5/h [2]; AHI < 10/h with ≥50% AHI improvement [3]; ≥50% AHI improvement. There was no significant difference in the response rates between MM and DISE. Abbreviations: MM, Muller’s maneuver; DISE, drug-induced sleep endoscopy; AHI, apnea-hypopnea index.

**Fig. 2:**
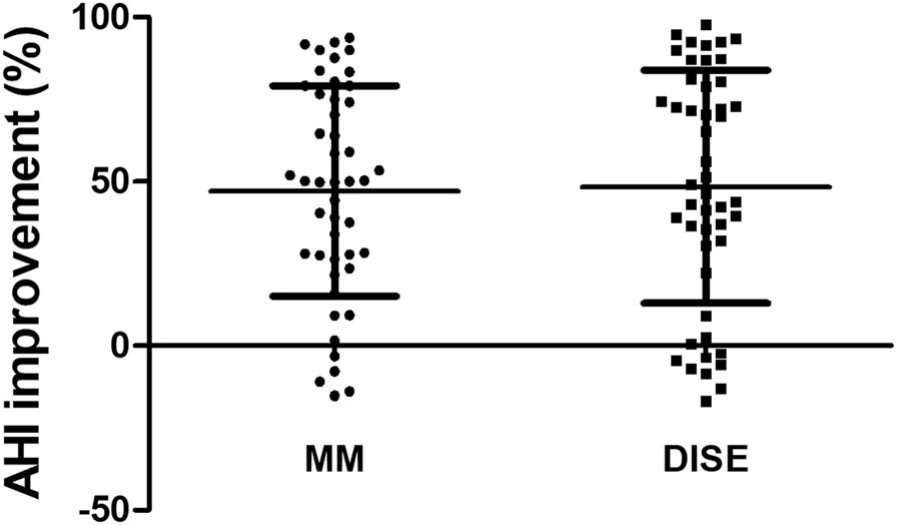
Comparison of AHI improvement after OSA surgery according to MM and DISE. There was no significant difference in AHI improvement between MM and DISE (*p* = 0.852). Abbreviations: AHI, apnea-hypopnea index; OSA, obstructive sleep apnea; MM, Muller’s maneuver; DISE, drug-induced sleep endoscopy.

The treatment response is presented in Fig. 1. Complete resolution (definition 1) was observed in 10.6% of patients in the MM group and in 16.7% in the DISE group (*p* = 0.393). The ratio of surgical success (definition 2) was 42.6% in the MM group and 45.8% in the DISE group (*p* = 0.748). The ratio of improved AHI ≥50% (definition 3) was 51.1% in the MM group and 47.9% in the DISE group (*p* = 0.759). However, these treatment responses were not significantly different between the groups. Improvement ratio of AHI was larger for patients in the DISE group (48.3 ± 35.4) than for those in the MM group (47.0 ± 32.0), but without statistical significance (*p* = 0.852) (Fig. 2).

**Fig. 3:**
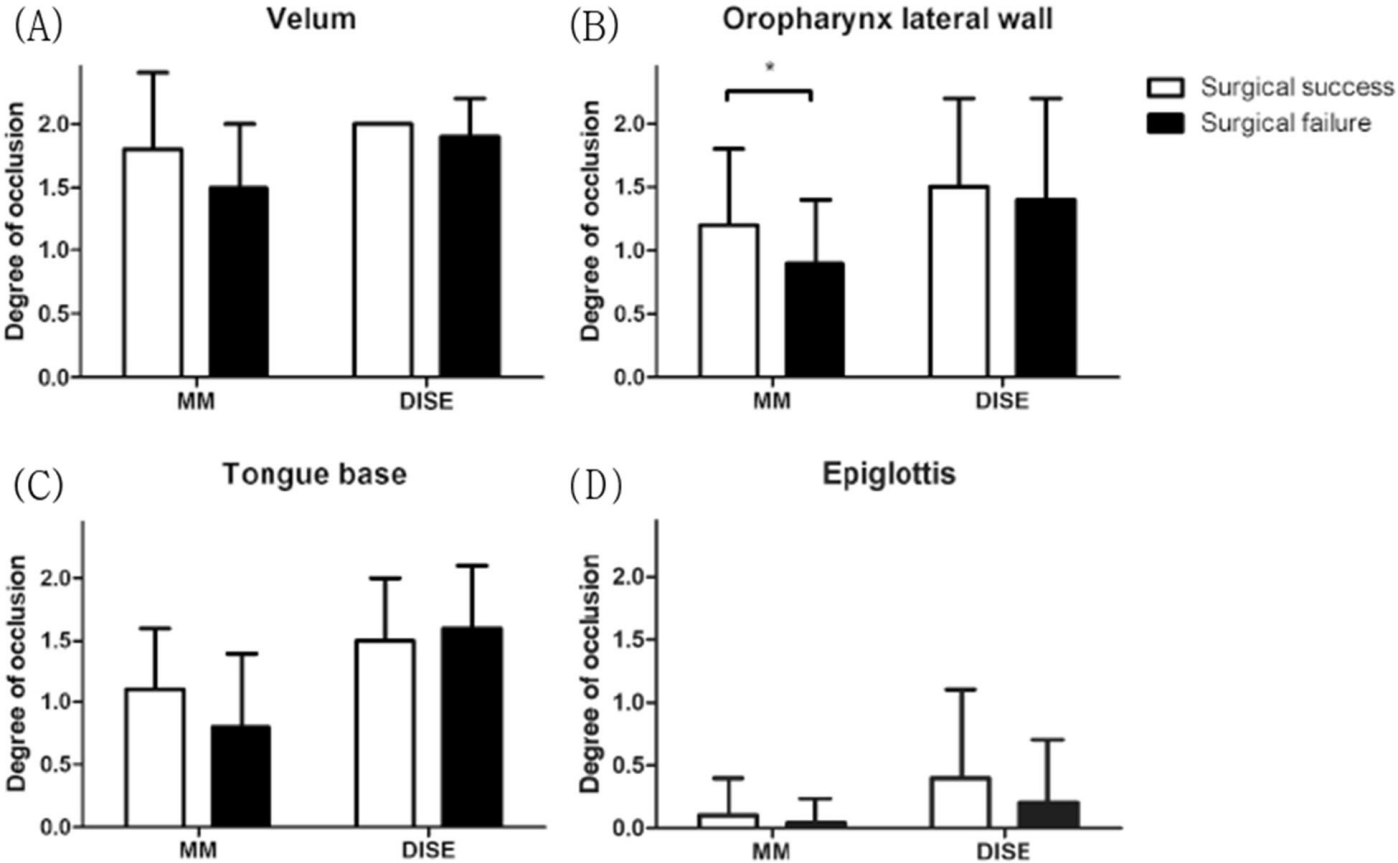
Comparison of the degree of occlusion between surgical success and surgical failure at each anatomic level: **a** velum, **b** oropharynx lateral wall, **c** tongue base, and **d** epiglottis. Oropharyngeal lateral wall occlusion assessed by MM in the MM group showed significant differences between surgical success and surgical failure (*p* = 0.031), whereas that assessed by DISE in the DISE group did not. Abbreviations: MM, Muller’s maneuver; DISE, drug-induced sleep endoscopy.

Results of identifying predictive factors that may affect surgical success are shown in Table 5. There was no significant difference between the surgical success and surgical failure in age, BMI, neck circumference, W/H ratio, modified Mallampati grade, Friedman stage, ESS, and several PSG parameters (such as AHI, supine AHI, AHI_NREM,_ AHI_REM,_ and nadir O_2_ sat). Tonsil grade was a significant predictive factor for surgical success in the analyses in both groups (*p* = 0.004 in the MM group, *p* = 0.042 in the DISE group). Anatomical occlusion level was assessed using nasopharyngoscopic study for surgical decision (MM for the MM group, DISE for the DISE group). Interestingly, occlusion of the oropharyngeal lateral wall assessed by MM in the MM group showed a significant difference between surgical success and failure (*p* = 0.031), whereas that assessed by DISE in the DISE group did not show a significant difference (*p* = 0.596). Other anatomical occlusion levels showed no significant differences between surgical success and failure (Fig. 3).

**Table 5:** Predictive values for surgical success

		MM group			DISE group		
		Surgical success (*n* = 20)	Surgical failure (*n* = 27)	*P -* value	Surgical success (*n* = 22)	Surgical failure (*n* = 26)	*P -*value
Sex, male (%)		15 (75%)	25 (92.6%)	0.094	20 (90.1%)	22 (84.6%)	0.511
Age		43.7 ± 11.2	44.8 ± 13.0	0.768	38.5 ± 10.4	44.5 ± 13.2	0.090
BMI		25.4 ± 2.8	25.5 ± 3.4	0.852	26.3 ± 3.5	26.3 ± 3.0	0.958
NC		40.1 ± 12.9	37.7 ± 2.5	0.371	39.0 ± 2.7	38.7 ± 3.2	0.703
W/H ratio		0.91 ± 0.06	0.92 ± 0.07	0.649	0.88 ± 0.07	0.92 ± 0.05	0.018*
Tonsil grade		1.9 ± 0.8	1.2 ± 0.7	0.004**	2.1 ± 0.9	1.6 ± 0.8	0.042*
MMP		3.0 ± 0.8	2.9 ± 0.7	0.670	3.1 ± 0.6	2.9 ± 0.9	0.583
Friedman staging		2.5 ± 0.8	2.8 ± 0.4	0.343	2.2 ± 0.6	2.4 ± 0.8	0.486
AHI		37.9 ± 2.4	41.0 ± 17.8	0.581	53.2 ± 21.7	43.7 ± 19.2	0.115
Supine AHI		53.6 ± 24.0	53.7 ± 21.4	0.996	66.0 ± 19.2	58.7 ± 23.0	0.259
AHI_NREM_		37.0 ± 19.0	41.9 ± 19.2	0.405	55.4 ± 22.7	45.0 ± 21.1	0.115
AHI_REM_		37.5 ± 29.2	33.4 ± 21.4	0.603	53.7 ± 25.3	46.8 ± 24.8	0.378
Nadir O_2_ sat, (%)		81.4 ± 4.8	79.4 ± 8.2	0.294	77.1 ± 8.7	78.3 ± 9.5	0.632
ESS		9.7 ± 5.1	9.4 ± 4.8	0.850	8.5 ± 5.2	7.8 ± 5.2	0.682
Nasopharyngoscopic evaluation ^a^	V	1.8 ± 0.6	1.5 ± 0.5	0.143	2.0 ± 0.0	1.9 ± 0.3	0.161
	O	1.2 ± 0.6	0.9 ± 0.5	0.031*	1.5 ± 0.7	1.4 ± 0.8	0.596
	T	1.1 ± 0.5	0.8 ± 0.6	0.112	1.5 ± 0.5	1.6 ± 0.5	0.275
	E	0.1 ± 0.3	0.04 ± 0.2	0.394	0.4 ± 0.7	0.2 ± 0.5	0.186
Nasal surgery, n (%)		11 (55.0%)	19 (70.3%)	0.278	11 (50%)	11 (42.3%)	0.594

*Abbreviations*: *MM* Muller’s maneuver, *DISE* Drug-induced sleep endoscopy, *BMI* Body mass index, *NC* Neck circumference, *W/H ratio* Waist/hip ratio, *MMP* Modified Mallampati grade, *AHI* Apnea-hypopnea index, *NREM* Non-rapid eye movement sleep, *REM* Rapid eye movement sleep, *Nadir O*_*2*_*sat* Nadir O_2_ saturation, *ESS* Epworth sleepiness scale, *V* Velum, *O* Oropharynx lateral wall, *T* Tongue base, *E* Epiglottis

* *p* < 0.05, **p < 0.01; statistical analyses were performed by independent t-test

^a^ Scoring occlusion level was represented according to the surgical decision methods for each group; by MM for MM group and by DISE for DISE group

## Discussion

In this study, our aim was to compare the outcomes of tongue base surgery based on MM and DISE in OSA patients. Furthermore, we tried to identify factors predictive of surgical success in nasopharyngoscopic study.

Identification of the airway obstruction site is important to determine the method of surgical treatment in OSA patients. OSA patients can exhibit multilevel obstruction, including those at the nasal and retrolingual levels. In 1984, Fusita [19] firstly described different anatomic levels of obstruction in OSA patients. He pointed out that most nonresponders to UPPP have multilevel obstruction, such as combined oropharyngeal and hypopharyngeal segments. Outcomes of sleep surgery are affected by the obstruction site and surgical techniques [18, 20, 21].

Surgical planning mainly depends on the identification of the site and degree of obstruction. Endoscopic examination has been considered as an indispensable technique to identify the exact site of obstruction in OSA patients. Awake fiber-optic nasal endoscopic evaluation with MM has been performed commonly at outpatient clinics. However, it is considered less effective in predicting surgical outcomes because it tends to underestimate the collapse at the retrolingual level and cannot assess the actual state of sleep [22, 23]. To overcome these shortcomings, DISE has been widely used over the last 20 years. Several recent studies have tried to identify concordance between the MM and DISE findings. These studies have reported significant concordance between MM and DISE for evaluating obstruction at the retropalatal level and lateral wall of the oropharynx, but the results for obstruction at the tongue base level were discordant between MM and DISE [7, 8]. Moreover, these studies also reported that MM has an increased tendency of underestimating obstruction compared with DISE. Our data also showed weak concordance at the tongue base level, and the tendency of MM to underestimate (Tables 2 and 3). From these results, one might expect that DISE would be more sensitive than MM, especially for detecting occlusion at the tongue base level.

Several studies have shown the impact of DISE in planning the surgical method and its outcome in OSA patients. As we have mentioned above, some recent studies have reported that surgical plan based on awake examination was changed after performing DISE in 40–50% of patients [9, 10]. There are several conflicting reports about the surgical outcomes of DISE. Some studies have reported that DISE-based diagnosis increases the success rates of sleep surgery [24, 25]. In a multicenter study on OSA patients with single- or multi-level occlusion, the surgical outcome of DISE was significantly worse than that of MM [26]. However, most of these previous studies compared the surgical outcomes based on MM and DISE, with respect to UPPP (including tonsillectomy), and did not focus on the tongue base surgery [13]. In this study, we tried to obtain results in a more controlled population by including only patients with multilevel occlusion.

As per our findings, the success rate of tongue base surgery based on DISE was slightly higher than that of surgery based on MM; however, the difference was not statistically significant. Results of the AHI reduction ratio in patients after the tongue base surgery based on both techniques were also not significantly different. There are several possible reasons why the surgical outcomes were not statistically different between the MM and DISE groups. First, DISE was basically recommended for patients with a mismatch on MM and PSG results in our study, indicating that patients in the DISE group might have more complex OSA than those in the MM group. Although there was no significant difference in demographic data between the MM and DISE groups (Table 1), there is a potential for selection bias that can lead to these negative consequences. Second, DISE does not reflect the actual sleep state of the airway as it changes the sleep architecture. For example, only non-REM sleep status is generally attained by DISE [27]. Moreover, the duration of examination is not identical to that of the natural physiological sleep. Third, tongue base resection surgery might not effectively improve the airway obstruction at the tongue base level. It has been expected that DISE can discriminate occlusion of the tongue base more sensitively and with a higher quality than MM, which can lead to a better surgical outcome. However, according to our results, there were no significant differences in the surgical outcomes between the two methods. Because the indication of tongue base surgery has not been strictly established, it may be necessary to clarify the criteria for patient selection to improve the surgical outcomes. Finally, there is also a possibility that the evaluative method or grading system used in the DISE and MM tests is not elaborate and may not accurately reflect the collapsible pattern or location. Judging from our results and other those of previous studies, there is still not enough evidence to conclude that surgical results of DISE are superior to those of the awake test.

In the present study, the tonsil size was statistically related to surgical success. Interestingly, the mean value of MM findings on the oropharyngeal lateral wall occlusion was significantly different between surgical success and surgical failure, whereas the mean value of DISE findings was not. The degree of occlusion of the oropharyngeal lateral wall can be generally considered to be related to the tonsil size. Therefore, these findings of MM on the oropharyngeal lateral wall seem reasonable. This mismatch between the MM and DISE findings suggests that the findings of DISE might be more exaggerated in measuring occlusion of the oropharyngeal lateral wall than those of MM.

There were also some limitations to our study. This study was retrospective in nature, so it might not have been as well controlled as a prospective study. Moreover, there was no statistical difference in surgical outcomes between the MM and DISE groups. This may be attributed to the small number of patients that were enrolled in this study. Therefore, prospective studies are needed in the future to more accurately evaluate the therapeutic value of DISE in tongue base surgery.

## Conclusions

This study was conducted to compare the surgical outcomes of MM and DISE, which are widely used to determine sleep surgery. From this study on the tongue base surgery, MM and DISE findings showed low agreement. DISE might affect the surgical decision in OSA patients compared to MM. However, there was no significant difference in the surgical results between MM and DISE. However, because this study is of the limited outcome under a retrospective design, there is not enough evidence to conclude on the comparison of surgical outcomes between MM and DISE. We look forward to a follow-up study comparing the surgical outcomes of MM and DISE under a prospective design.

## Data Availability

The datasets analyzed during the current study are available from the corresponding author on reasonable request.

## Abbreviations

AHI: Apnea-hypopnea index
BMI: Body mass index
CPAP: Continuous positive airway pressure
DISE: Drug-induced sleep endoscopy
E: Epiglottis
ESS: Epworth sleepiness scale
LP: Lateral pharyngoplasty
MM: Muller’s maneuver
O: Oropharynx lateral wall
OSA: Obstructive sleep apnea
PSG: Polysomnography
REM: Rapid eyeball movement
T: Tongue base
UPPP: Uvulopalatopharyngoplasty
V: Velum
